# CT-assessment of carotid plaque features and their impact on residual stenosis after stenting

**DOI:** 10.3389/fneur.2026.1767502

**Published:** 2026-03-04

**Authors:** Lu Li, Ting Ting Li, Qing Yuan Wang, Yu Meng Sun, Zhen Jia Wang, Su Nan Xu, Wei Yu

**Affiliations:** 1 Department of Radiology, Beijing Anzhen Hospital, Capital Medical University, Beijing, China; 2 Department of Vascular Surgery, Beijing Anzhen Hospital, Capital Medical University, Beijing, China

**Keywords:** calcium, carotid atherosclerosis, plaque component, residual stenosis, stenting

## Abstract

**Objectives:**

It is well established that calcified plaques are highly likely to lead to residual stenosis after stenting; however, the specific characteristics responsible for this effect remain unknown. This study aimed to identify both qualitative and quantitative imaging risk factors for residual stenosis using computed tomography angiography.

**Methods:**

We retrospectively enrolled 233 patients with carotid artery stenosis. Patients were categorized into two groups based on the presence or absence of postoperative residual stenosis. Carotid computed tomography angiography evaluated plaque characteristics both qualitatively and quantitatively. Logistic regression analysis identified independent risk factors for residual stenosis. We evaluated the predictive model’s discriminative ability by calculating the area under the receiver operating characteristic (ROC) curve.

**Results:**

Univariate analysis indicated a statistical difference in age, creatinine, total plaque volume, percentage of total calcified plaque, percentage of total soft plaque, maximum slice attenuation value, maximum thickness, total length, and a circumferential calcification score ≥2 points (*p* < 0.05). Multivariable logistic regression identified creatinine (OR = 1. 020; 95%CI: 1.005–1.035; *p* = 0.010), maximum slice attenuation value(Z-score; OR = 1.627; 95%CI: 1.024–2.585; *p* = 0.039), percentage of calcified plaque volume(Z-score; OR = 1.872; 95%CI: 1.137–3.082; *p* = 0.014) and circumferential calcification score ≥2 (OR = 3.257; 95%CI: 1.620–6.548; *p* < 0.001) as independent factors associated with residual stenosis. Furthermore, receiver operating characteristic curve analysis revealed that the area under the curve for the combined model in diagnosing residual stenosis was 0.784.

**Conclusion:**

In conclusion, preoperative CTA-based assessment of specific plaque characteristics, such as calcified plaque volume percentage, circumferential calcium score, and the maximum slice attenuation value of calcification are related to residual stenosis.

## Introduction

Carotid artery stenting (CAS) is currently a commonly used surgical approach for the treatment of carotid atherosclerotic stenosis (1). Post-stenting residual stenosis is a strong predictor of in-stent restenosis, one of the most significant complications associated with the procedure (2, 3). A recent study reported an overall incidence of residual stenosis at 22.8% (130/570 stents). Furthermore, 13% of the patients with residual stenosis developed restenosis during the follow-up period (4).

Plaque characteristics are critical determinants of procedural outcomes (5). Low-density plaque is associated with an increased risk of postoperative stroke, whereas calcified plaque is considered a protective factor. Severe calcification poses a challenge to adequate stent expansion (6, 7). Although numerous studies have confirmed calcification as the primary factor in residual stenosis post-stenting, only a few have explored the detailed characteristics of carotid plaque components and their correlation with residual stenosis (5, 7). Therefore, it remains unclear which calcified plaque features detected by CTA contribute to residual stenosis.

This study aimed to assess plaque features using CTA to determine independent predictors of residual stenosis following carotid stenting.

## Materials and methods

### Study population and study design

**Figure 1:**
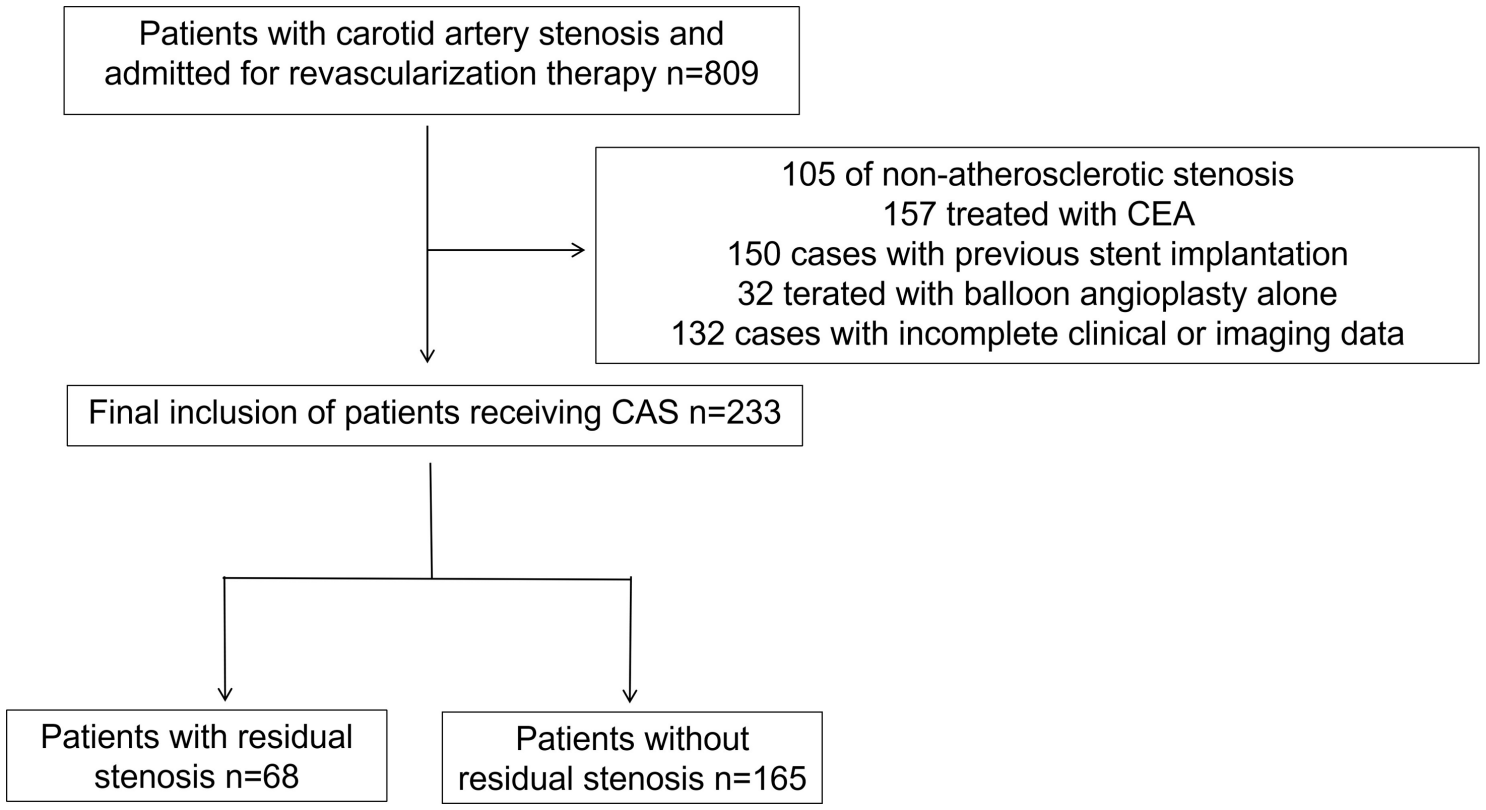
Flow chart of the study. CAS, carotid stenting; CEA, carotid endarterectomy.

The study was conducted retrospectively in accordance with the Declaration of Helsinki (as revised in 2013). Patients with angiographically confirmed carotid artery stenosis who underwent revascularization therapy between January 2022 to January 2024. Inclusion criteria comprised: (1) patients with symptomatic carotid stenosis ≥ 50% or asymptomatic carotid stenosis ≥ 70% according to the NASCET criteria; (2) individuals undergoing primary carotid revascularization; (3) patients who underwent carotid CTA within 1 week prior to CAS. The exclusion criteria included the following: (1) non-atherosclerotic carotid stenosis, such as carotid artery dissection, fibromuscular dysplasia, Moyamoya disease, and vasculitis; (2) treated with CEA. (3) Patients with incomplete clinical or imaging data. A flowchart detailing the study’s progression is shown in Figure 1.

Patients were stratified into two cohorts based on the presence or absence of postprocedural residual stenosis, as confirmed by digital subtraction angiography (DSA) following CAS. Retrospective analysis included demographic characteristics (age, sex), comorbidities (hypertension, diabetes mellitus, hyperlipidemia, history of coronary artery disease and stroke), systolic and diastolic blood pressure, and lifestyle factors (smoking and drinking). Admission laboratory indices included white cell count, creatinine, hcy, hs-CRP, glucose, and lipids (TG, TC, triglycerides, HDL-C, LDL-C). Imaging parameters comprised the preoperative luminal stenosis rate, total plaque volume, plaque remodeling index and eccentricity index, the presence of ulceration, the percentage of total volume occupied by calcified and non-calcified plaques, and characteristics of calcified plaques.

### Imaging analysis

#### CTA imaging techniques and analysis

The CTA examinations were performed with a consistent protocol, ensuring the scan encompassed from the aortic arch to the apex of the cranial vault. Access was established intravenously through the median cubital vein at the anterior elbow, with contrast medium administered at 4 mL/s until a total of 100 mL was infused; the vein was subsequently flushed with saline at the same rate until a total of 35 mL was administered. Scanning was initiated upon contrast arrival in the carotid artery.

Preoperative carotid CTA images of all patients were uploaded to the PACS system. CTA images were analyzed using commercial software (Linkage Intelligence, Shanghai, China) to derive the total plaque volume, volumes of the calcified and non-calcified plaques, and their volumes percentage. Calcified plaques were defined as plaques with attenuation values ≥ 350 Hu, whereas plaques with attenuation values < 350 Hu were defined as non-calcified plaques. Remodeling index (RI) was calculated as the vessel area at the site of maximal stenosis divided by the vessel area at the reference segment. The eccentricity index was calculated as (maximum plaque thickness–minimum plaque thickness) / maximum plaque thickness. The presence of ulceration was identified by contrast agent extending ≥1 mm beyond the luminal border (8). The region of interest (ROI) of the most calcified plaque was manually outlined to measure its attenuation. The maximum wall thickness, total calcification length, and maximum calcification thickness were manually measured. The preoperative degree of stenosis was measured using NASCET criteria (9), as follows: preoperative stenosis rate (%) = (1–diameter at the narrowest point/normal diameter) × 100%.

**Figure 2:**
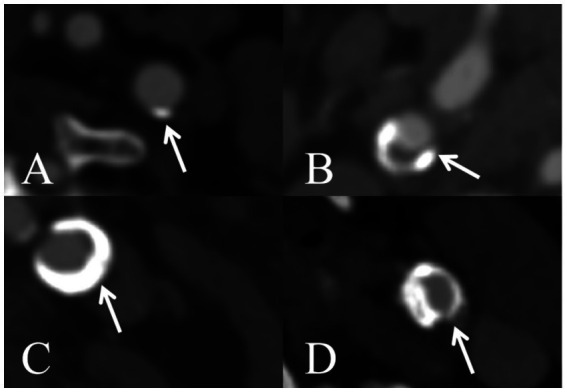
Schematic illustration of the degree of axial calcification distribution in the carotid arteries **(A)** calcification circumference < 90°, **(B)** calcification circumference 90–180°, **(C)** calcification circumference 180–270°, and **(D)** calcification circumference ≥270°.

We applied the same criteria as the previous study to assess the maximum circumferential distribution of calcification (10). The distribution of carotid artery calcification was assessed using a 5-point scoring system: 0, none; 1, circumferential degree < 90°; 2, circumferential degree 90°–180°; 3, circumferential degree 180°–270°; and 4, circumferential degree ≥ 270° (Figure 2).

#### CAS implantation and analysis

The procedure was performed as outlined in the 2023 European Society for Vascular Surgery (ESVS) clinical practice guidelines for the management of atherosclerotic carotid artery disease (11). Prior to the procedure, all patients received a standard 3-day regimen of aspirin (100 mg/day) plus either clopidogrel (75 mg/day) or ticlopidine (250 mg/day). All procedures were performed under local anesthesia by experienced surgeons. The intervention was initiated by fluoroscopically identifying the site of maximal luminal narrowing, followed by the advancement of a cerebral protection device (SpiderFx,ev3, Plymouth, MN, United States, or Filterwire EZ, Boston Scientific, Natick, MA, United States) and pre-dilation with an angioplasty balloon(the Sterling™ balloon catheter, Boston Scientific, Marlborough, MA, United States). At the stenotic site, all patients received either an open-cell stent (PRECISE PRO RX, Cordis; or Protege RX, ev3/Medtronic) or a closed-cell stent (Wallstent, Boston Scientific). After stent expansion, angiographic reassessment was performed, and post-stenting balloon angioplasty was instituted for any residual stenosis greater than 50%. The balloon inflation pressure was typically set at the nominal level, approximately 6–8 atm.

Postoperative residual stenosis was measured following stent deployment. If balloon post-dilation was performed, the measurement was taken before the dilation procedure. Postoperative residual stenosis was assessed by DSA, defined as ≥30% stenosis (7). The calculation formula was applied to determine the degree of the stenosis as follows: Post-stent residual stenosis (%) = (1–in-stent diameter/normal diameter) × 100%.

### Statistical analysis

Data normality was assessed with the Shapiro–Wilk test. Normally distributed continuous variables are reported as mean ± SD; non-normal data as median (IQR); and categorical variables as n (%). Intergroup comparisons were performed using Student’s t-test for normally distributed continuous variables or the Mann–Whitney U test for nonparametric data, with χ^2^ tests employed for categorical variables. Variables demonstrating statistical significance (*p* < 0.05) were incorporated into a binary logistic regression analysis. Standardization was performed on the continuous variables that exhibited non-normal distributions, transforming them into Z-scores for subsequent inclusion in the regression analysis. Receiver operating characteristic (ROC) curve analysis was performed to assess the predictive performance of identified independent risk factors for residual stenosis. The discriminatory capacity of different predictive models was evaluated by comparing the areas under the ROC curves (AUC). Two-sided *p* < 0.05 indicated statistical significance. All statistical analyses were performed using commercial SPSS software (IBM SPSS Statistics for Windows, Version 25.0; IBM Corp, Armonk, NY).

## Results

### Baseline characteristics of patients

Table 1 presents the baseline demographic and clinical characteristics of the study population. A total of 233 patients were enrolled in the study, meeting the inclusion and exclusion criteria. The cohort comprised 196 males (84.1%), with a mean age of 68.3 ± 7.0 years. Postprocedural evaluation revealed residual stenosis in 68 cases (29.2%).

**Table 1:** Baseline characteristics of the study population.

Characteristics	With residual stenosis (*n* = 68)	No residual stenosis (*n* = 165)	*p*
Age (y)	69.8 ± 6.0	67.7 ± 7.2	0.039*
Male, n (%)	53 (77.9)	143 (86.7)	0.098
Hypertension, n (%)	54 (79.4)	123 (74.5)	0.429
Diabetes, n (%)	26 (38.2)	60 (36.4)	0.350
History of coronary artery disease, n (%)	26 (38.2)	46 (27.9)	0.160
History of stroke, n (%)	13 (19.1)	35 (21.2)	0.720
SBP (mmHg)	130 (120–139.5)	134 (125–144)	0.050
DBP (mmHg)	75 (65–82.5)	78 (10–81)	0.224
WBC (×10^9^/L)	6.36 (5.48–7.64)	6.39 (5.59–7.75)	0.695
Creatinine (μmol/L)	80.95 (70.75–99.1)	74.3 (66.7–84.5)	0.019*
Hcy (μmol/L)	14.65 (11.25–20.08)	14.3 (11.8–19.2)	0.914
hs-CRP (mg/L)	1.24 (0.72–3.42)	1.22 (0.70–3.96)	0.469
Glucose (mmol/L)	5.52 (4.92–6.81)	5.55 (4.75–6.85)	0.393
TG (mmol/L)	1.38 (0.97–1.83)	1.27 (0.92–1.77)	0.494
TC (mmol/L)	3.81 (3.13–4.32)	3.62 (3.18–4.34)	0.387
HDL-C (mmol/L)	1.06 (0.88–1.25)	0.99 (0.85–1.19)	0.182
LDL-C (mmol/L)	1.99 (1.64–2.48)	1.95 (1.63–2.41)	0.803
Smoking, n (%)	43 (63.2)	112 (67.9)	0.542
Drinking, n (%)	25 (36.8)	77 (46.7)	0.192
Open-cell stents, n (%)	19 (27.9)	58 (35.2)	0.083
Post-dilation balloon, n (%)	14 (20.6%)	17 (10.3%)	0.034*

SBP, systolic blood pressure; DBP, diastolic blood pressure; WBC, white cell count; Hcy, homocysteine; hs-CRP, high-sensitivity C-reactive protein; TG, triglyceride; TC, total cholesterol; HDL, high-density lipoprotein; LDL, low density lipoprotein. Data are expressed as mean ± SD or n (%). *p < 0.05 was considered statistically significant.

Sex, hypertension, diabetes, hyperlipidemia, history of coronary artery disease and stroke did not differ significantly between patients with versus without residual stenosis. Systolic and diastolic blood pressure, white blood cell count(WBC), homocysteine, high-sensitivity C-reactive protein (hs-CRP), fasting blood glucose, and lipid level were also similar. Age (*p* = 0.039) and serum creatinine level (*p* = 0.019) differed between the groups: the residual stenosis group was older and had higher creatinine level. Post-dilation balloon angioplasty was performed more frequently in the group with residual stenosis (*p* = 0.034). Smoking, drinking and stent type were not significantly different.

### CTA imaging features and comparison between groups

**Figure 3:**
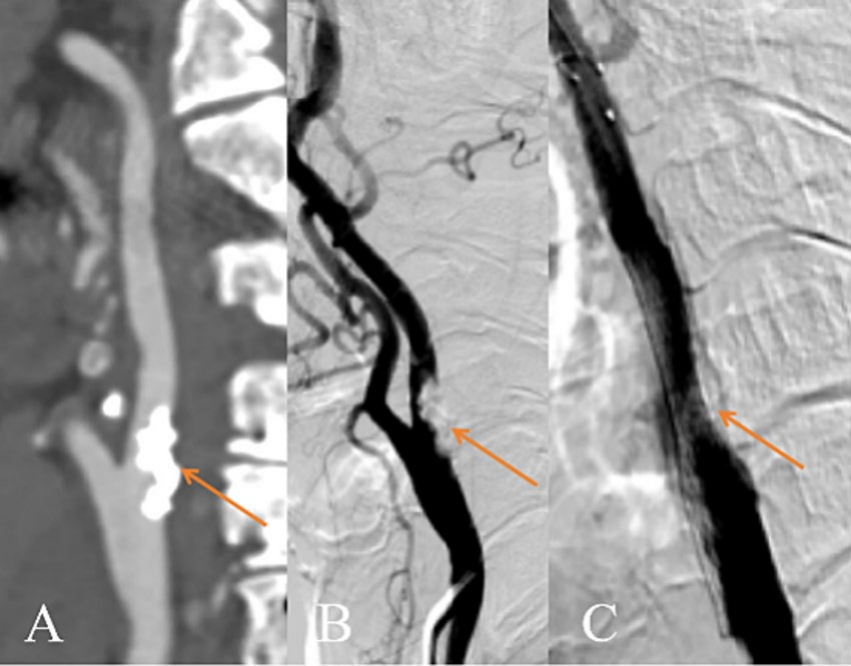
Diagram of the internal carotid artery with residual stenosis after CAS surgery. **(A)** CTA shows calcified plaque formation in the proximal segment of the left internal carotid artery with severe luminal stenosis (shown by the orange arrow); **(B)** Preoperative DSA of the CAS shows severe stenosis of the proximal segment of the left internal carotid artery (shown by the orange arrow); **(C)** Postoperative DSA showing poor expansion of an open-loop stent in the proximal segment of the left common carotid artery (shown by the orange arrow). CTA, Computer tomography angiography. DSA, Digistal subtraction angiography. CAS, Carotid artery stenting.

**Figure 4:**
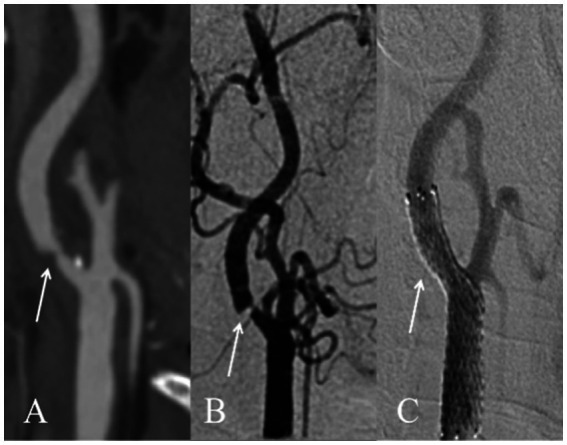
Diagram of the internal carotid artery without residual stenosis after CAS surgery. **(A)** CTA demonstrates the presence of mixed plaque formation in the proximal segment of the right internal carotid artery, accompanied by significant luminal stenosis (indicated by the white arrow); **(B)** Preoperative DSA reveals critical stenosis in the proximal segment of the right internal carotid artery (indicated by the white arrow); **(C)** Postoperative DSA imaging confirms optimal deployment of an open-cell stent in the proximal segment spanning from the right common carotid artery to the internal carotid artery (indicated by the white arrow), demonstrating complete vessel patency. CTA, Computer tomography angiography. DSA, Digital subtraction angiography.

Univariate analysis of CTA imaging features between patients with and without residual stenosis is presented in Table 2. Between residual stenosis and without residual stenosis groups, there were no significant differences in preoperative lumen stenosis rate, maximum wall thickness, remodeling index, plaque eccentricity index, or the prevalence of ulceration. The group with residual stenosis exhibited significantly larger total plaque volume (1198.6 mm^3^ vs. 821.8 mm^3^, *p* = 0.005) and a higher percentage of calcified plaque volume (17.5% vs. 9.0%, *p* < 0.001), while demonstrating a lower percentage of non-calcified plaque (82.5% vs. 91.0%, *p* < 0.001). Specifically, calcified plaques in the residual stenosis group showed higher maximum attenuation value (802.0 HU vs. 583.0 HU, *p* < 0.001), greater maximum thickness (4.68 mm vs. 1.6 mm, *p* = 0.017), and longer total length (11.8 mm vs. 9.0 mm, *p* = 0.008). Furthermore, circumferential calcification scoring at the narrowest lumen slice demonstrated a significant association with residual stenosis, with a higher proportion of scores ≥2 points in the residual stenosis group (58.8% vs. 32.1%, *p* < 0.001). Representative DSA and CTA findings are illustrated in Figures 3, 4, respectively.

**Table 2:** Comparison of CTA imaging features in the group with and without residual stenosis.

Variables	With residual stenosis (*n* = 68)	No residual stenosis (*n* = 165)	*p*
Preoperative lumen stenosis rate (%)	72.3 ± 11.1	70.7 ± 10.1	0.277
Total plaque volume (mm^3^)	1198.6 (632.6–1840.1)	821.8 (437.7–1342.4)	0.005*
Percentage of calcified plaque volume (%)	17.5 (8–43.1)	9 (2–21)	<0.001*
Percentage of soft plaque volume (%)	82.5 (56.9–92.0)	91 (78.5–98)	< 0.001*
Maximum wall thickness (mm)	2.0 (1.43–2.68)	4.5 (3.7–5.6)	0.732
Remodeling index	1.5 (1.0–1.8)	1.3 (1.0–2.0)	0.934
Plaque eccentricity index	0.83 (0.65–0.90)	0.81 (0.65–0.89)	0.524
Ulceration	12 (17.6)	42 (25.5)	0.234
Calcified plaque features			
Maximum slice attenuation value (Hu)	802.0 (598.5–1027.0)	583 (364.5–765.5)	< 0.001*
Maximum thickness (mm)	4.68 (3.7–5.7)	1.6 (1.1–2.4)	0.017*
Total length (mm)	11.8 (6.3–16.7)	9 (3.8–13.5)	0.008*
Grouping of circumferential calcification score at lumen’s narrowest point, n (%)			< 0.001*
<2 points	28 (41.2%)	112 (67.9%)	
≥2 points	40 (58.8%)	53 (32.1%)	

Data are expressed as mean ± SD or n (%). *p < 0.05 was considered statistically significant.

### Risk factors for residual stenosis

Variables with a significance level of *p* < 0.05 in the univariate analysis were included in the binary logistic regression model. In multivariable logistic regression, serum creatinine level (OR = 1. 020; 95%CI: 1.005–1.035; *p* = 0.010), maximum slice attenuation value (Z-score; OR = 1.627; 95%CI: 1.024–2.585; *p* = 0.039), percentage of calcified plaque volume (Z-score; OR = 1.872; 95%CI: 1.137–3.082; *p* = 0.014) and circumferential calcification score ≥2 (OR = 3.257; 95%CI: 1.620–6.548; *p* < 0.001) were independently associated with residual stenosis. Age, total plaque volume, percentage of soft plaque volume, calcification thickness and total length did not differ significantly (Table 3). ROC curve analysis was performed to evaluate the predictive utility of independent variables and composite factors for residual stenosis. The calcification circumferential score, maximum slice attenuation value, and calcified plaque volume percentage all demonstrated moderate discriminatory ability, with AUC of 0.634 (95% CI: 0.554–0.713), 0.678 (95% CI: 0.602–0.755), and 0.679 (95% CI: 0.602–0.777), respectively. Based on the Youden index, a threshold of 40.6% was calculated to divide patients into low-risk and high-risk groups, with the latter exhibiting a higher incidence of residual stenosis (66.7% vs. 23.6%, *p* < 0.001).

**Table 3:** Binary logistic regression for residual stenosis.

Variable	β	SE	Wald $\chi^{2}$	*p*	OR	95% CI for OR
Age (y)	0.004	0.025	0.022	0.882	1.004	0.955–1.055
Creatinine ( μ mol/L)	0.019	0.008	6.649	0.010*	1.020	1.005–1.035
Total plaque volume (Z-score; mm^3^)	0.360	0.203	3.145	0.076	1.433	0.963–2.134
Percentage of calcified plaque volume (Z-score; %)	0.627	0.254	6.081	0.014*	1.872	1.137–3.082
Percentage of soft plaque volume (Z-score; %)	−0.375	3.330	0.013	0.910	0.687	0.001–469.609
Maximum slice attenuation value (Z-score; Hu)	0.487	0.236	4.251	0.039*	1.627	1.024–2.585
Calcification thickness (Z-score; mm)	−0.434	0.261	2.761	0.097	0.648	0.389–1.081
Total length (Z-score; mm)	−0.261	0.235	1.227	0.268	0.770	0.486–1.222
Narrowest point score ≥ 2	1.181	0.356	10.987	<0.001*	3.257	1.620–6.548

*p < 0.05 was considered statistically significant. β, unstandardized coefficient; SE, standard error; OR, odds ratio; CI, confidence interval.

**Figure 5:**
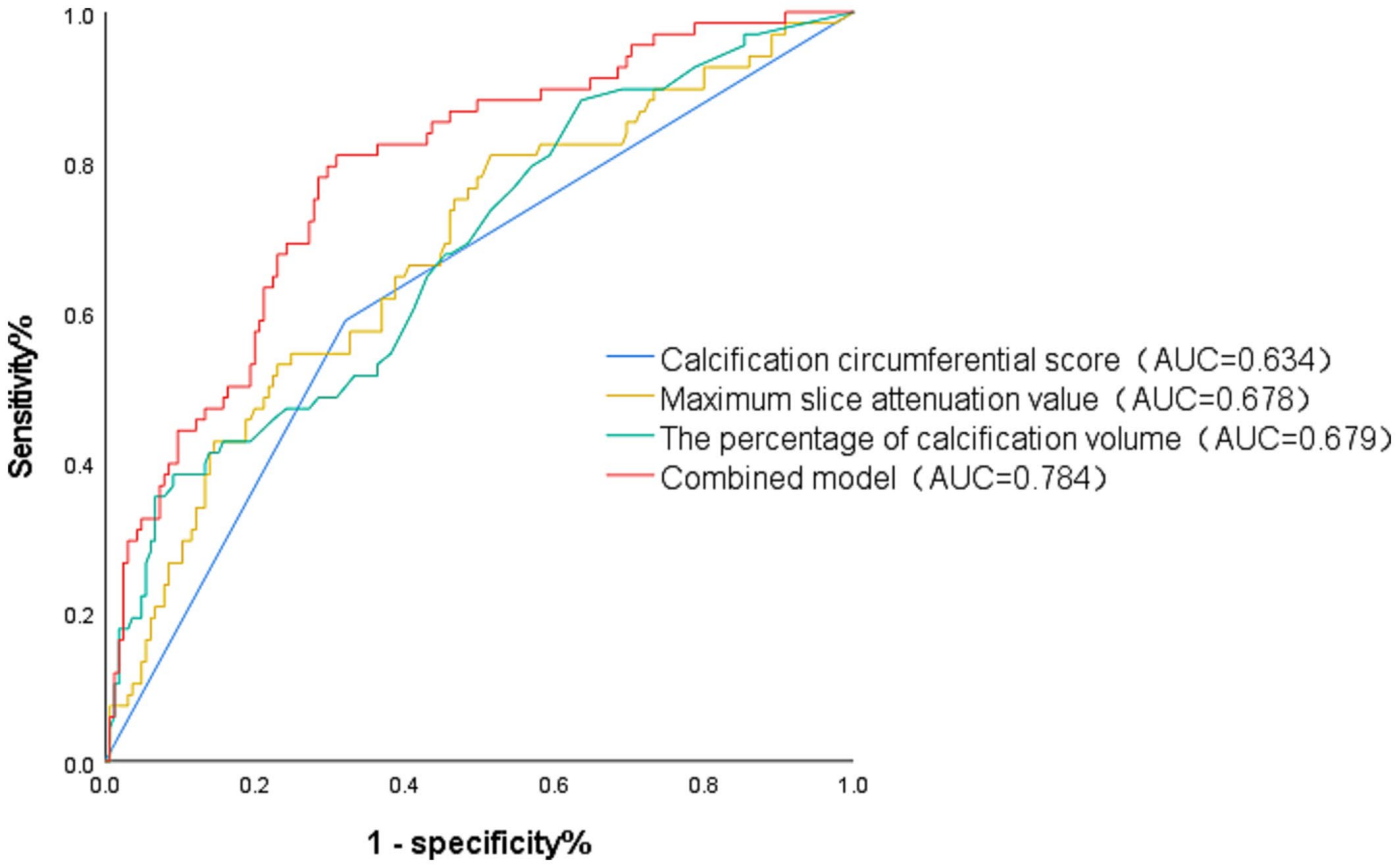
Results of the analysis of the ROC.

Building upon these findings, a multivariate logistic regression model incorporating these variables was developed. This combined model demonstrated moderate performance, achieving an AUC of 0.784 (95% CI: 0.72–0.848), with a sensitivity of 80.9% and a specificity of 69.1% (Figure 5).

## Discussion

The present study revealed that serum creatinine level, maximum slice attenuation value, the percentage of calcification volume, and circumferential calcification score ≥2 were independently associated with the postprocedural residual stenosis. The integration of these readily available features into a predictive model yielded a moderate discriminative ability (AUC = 0.784), suggesting its potential utility in identifying patients at high risk for postoperative residual stenosis.

Plaque characteristics are well-established determinants of inadequate stent expansion, with severe lesion calcification identified as a risk factor in previous studies (12, 13). The current research found that patients with residual stenosis after CAS exhibited more severe calcification characteristics than those without, including greater volume percentage, circumferential extent, length, and thickness (14).

Previous studies have indicated that stent expansion depends primarily on plaque compression and vessel wall stretching (15). When calcified plaque possesses sufficient rigidity to resist this expansion, they thereby cause residual stenosis (16). Higher attenuation calcified plaques, indicative of greater density and stiffness, are resistant to fracture during stent deployment, thereby restricting expansion and resulting in residual stenosis (17, 18). In our study, calcification circumference angle scores were dichotomized at a cutoff of 2 points, categorizing patients into high-score (score ≥ 2 points) and low-score (score < 2 points) groups. Multivariate analysis revealed that patients in the high-score group had 3.257-fold greater odds of residual stenosis compared to the low-score group (OR = 3.257; 95%CI: 1.620–6.548; *p* < 0.001). Similarly, Fujino A et al. (19) used optical coherence tomography (OCT) to quantitatively assess calcified plaque characteristics and found that a maximum calcification circumference of > 180° inhibited stent expansion. These findings indicate that calcification forms a continuous, circumferential structure. This configuration imposes uniform resistance around the entire stent circumference, which often prevents complete expansion despite post-dilation and consequently leads to residual stenosis (7). Therefore, when there is a higher proportion of calcification and the circumferential extent exceeds 180°, appropriate measures can be taken, such as preparing sufficient balloon dilation and increasing balloon pressure to reduce the occurrence of residual stenosis.

Previous research indicates differences in expansion properties between closed-cell and open-cell stents (10). However, our analysis showed no significant link between either stent type or residual stenosis. This may be attributable to the use of post-dilation balloon angioplasty in a subset of patients, who participated in the stent expansion process and mitigated a direct association between stent type and residual stenosis. Additionally, long-term follow-up revealed a higher rate of in-stent restenosis in the closed-cell stent group (20). The increased risk of restenosis is attributed to greater vessel wall irritation caused by the stiffer and denser material of closed-cell stents, leading to neointimal hyperplasia (21).

Although subjects in the non-residual-stenosis group were observed to have a higher percentage of non-calcified plaque (91% (78.5–98%)). As expected, non-calcified plaque was not identified as a risk factor for residual stenosis following CAS. This suggests that non-calcified plaques exert a weaker inhibitory effect on stent expansion, a finding consistent with prior observations (22). Increased stent elongation was observed in vessels with lower plaque density and greater wall distensibility (16, 23). Katano et al. (24) have demonstrated that lesions composed of severe calcium exhibit a significantly higher incidence of residual stenosis following CAS compared to those primarily consisting of lipids. A recent study based on high-resolution wall imaging discovered that the lipid necrotic core and intraplaque hemorrhage (IPH) had a minimal effect on stent expansion (25). Although the lipid necrotic core and IPH did not correlate with stent expansion, it has been demonstrated that patients with IPH experienced a higher rate of asymptomatic ischemia and a composite outcome of periprocedural stroke, death, and myocardial infarction (26). During carotid stenting, non-calcified plaque can embolize, potentially causing distal vessel occlusion and cerebral ischemia (27).

This study has several limitations. Firstly, this was a single-center retrospective study and there was a relatively small number of cases. The selection of stent types was subject to clinical selection bias. Second, the specificity of the combined model is only 69.1%, indicating that approximately 30% of patients classified as high-risk may not actually develop significant residual stenosis. This could result in unnecessary intensive follow-up, increasing the medical burden and patient anxiety. Future studies should further optimize feature selection or incorporate higher-resolution imaging markers (such as plaque microstructural features or hemodynamic parameters) to enhance specificity while maintaining high sensitivity. Third, the analysis was restricted to immediate postprocedural outcomes, with no longitudinal assessment of mid- or long-term results. Further investigation is required to evaluate the association between calcification features and in-stent restenosis following CAS, which may inform optimal revascularization strategy selection.

## Conclusion

From the perspective of plaque distribution imaging, our findings provide further evidence for the mechanism by which calcified plaques contribute to residual stenosis. To determine an appropriate treatment strategy for carotid artery stenosis with calcified plaques, assessment may include not only the near-circumferential distribution of calcification but also its volume percentage and stiffness. Future studies that incorporate long-term clinical follow-up will further clarify the impact of specific calcified plaque characteristics on adverse outcomes after stent placement, thereby offering more precise imaging guidance for personalized carotid revascularization.

## Data Availability

The raw data supporting the conclusions of this article will be made available by the authors, without undue reservation.
